# Scalp In-Transit Metastatic Melanoma Treated with Interleukin-2 and Pulsed Dye Laser

**DOI:** 10.3390/healthcare1010096

**Published:** 2013-10-25

**Authors:** Michael Z. Wang, Jerry D. Brewer

**Affiliations:** Department of Dermatology, Mayo Clinic, 200 First Street SW, Rochester, MN 55905, USA; E-Mail: wang08380@gmail.com

**Keywords:** interleukin-2, melanoma, pulsed dye laser therapy

## Abstract

No particular regimen is considered standard therapy for widespread metastatic melanoma, although surgery is the primary choice for regional nodal metastases. Systemic interleukin-2 (IL-2) is an effective immunotherapy for melanoma, but standard doses are associated with severe toxicity. We report a patient who was treated with intralesional low-dose IL-2 and V-beam pulsed dye laser for the treatment of scalp melanoma metastases. This treatment resulted in rapid regression of metastatic tumors with limited adverse effects.

## 1. Introduction

No particular regimen is considered standard therapy for widespread metastatic melanoma, and effectiveness of single-agent interventions has been questioned [1]. Here, we report a new approach with intralesional interleukin-2 (IL-2) and V-beam pulsed dye laser for the treatment of scalp melanoma metastases. 

## 2. Case Report

The patient was a 61-year-old man. Twenty-two years ago, a primary melanoma was removed from his neck. Eleven years later, a second primary melanoma developed on his scalp, and was surgically excised. Seven years later, a third melanoma (superficial spreading type) developed on the scalp (Clark level III, Breslow thickness 0.9 mm, and mitotic rate 2/mm^2^), and was treated with wide local excision and sentinel lymph node biopsy. Two lymph nodes harvested were negative. Half a year later, multiple in-transit metastases appeared on his scalp without signs of distant metastases. The lesions were surgically excised. However, multiple in-transit metastases continued to develop in the region rapidly. Thereafter, surgical excision and topical imiquimod 5% were initiated resulting in complete resolution of one lesion, whereas two others persisted.

**Figure 1:**
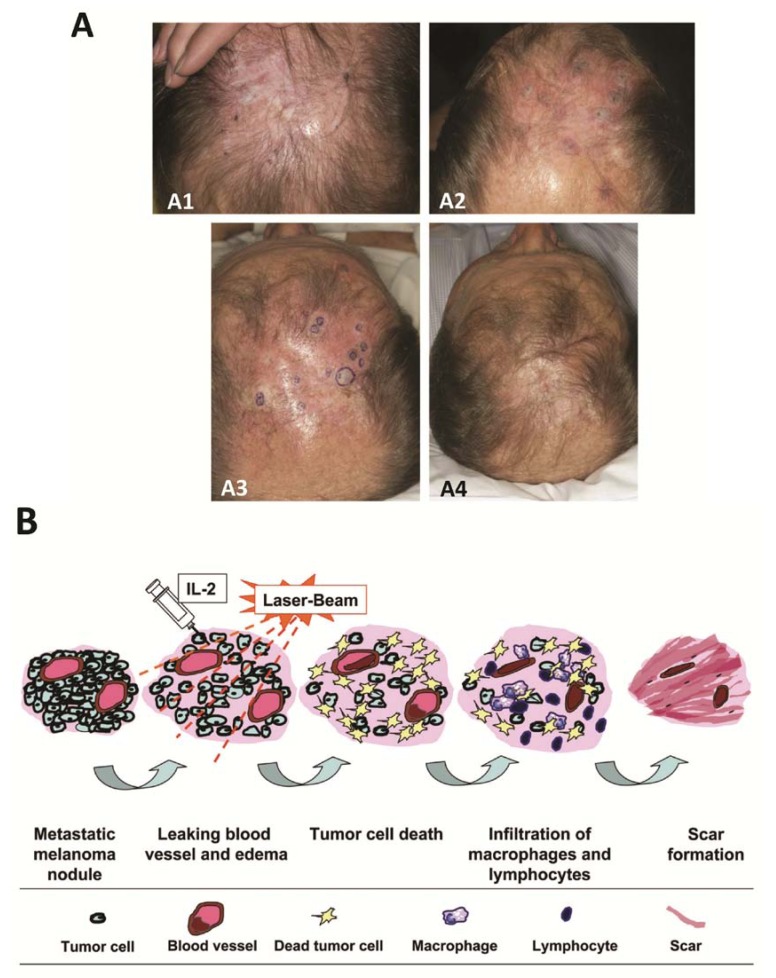
(**A1**) In-transit metastasis of melanoma at presentation (referral to our institution); (**A2**) After initial treatment with pulsed dye laser; (**A3**) At the initiation of interleukin-2 injections, in conjunction with continued pulsed dye laser therapy; (**A4**) Four and a half weeks after the addition of interlesional interleukin-2; (**B**) Possible mechanisms of the synergetic effects of intralesional interleukin-2 (IL-2) and local laser therapy.

The patient was referred to our institution (Figure 1A). The melanoma was genetically wild-type. Treatment began with systemic granulocyte-macrophage colony-stimulating factor (GMCSF) (250 mcg/day; alternating between 2 weeks of treatments and cessations), topical imiquimod 5% (twice/day), V-beam pulsed dye laser (every 2–4 weeks; 595 nm; 10-mm spot size), and periodic punch excisions from June through November 2010. Despite improvement, new lesions continued to develop. Intralesional IL-2 injections (6 million IUs, 2–3 times/week) were then added to the therapeutic regimen. Subsequently, within 1 month, all the tumors vanished (Figure 1A) with pathological confirmation. No severe toxicity was observed, and patient only had flu-like symptoms. Treatment was discontinued in December 2010. The patient remained clinically melanoma-free until July 2011, when a new solitary lesion appeared on vertex scalp. The 0.5 × 0.5-cm lesion was a freely mobile, subcutaneous nodule and was confirmed to be metastatic melanoma. IL-2 injections resumed with the previous protocol for a longer period, and complete response was achieved. By September 2011, biopsy showed no residual cancer in the area. 

## 3. Results and Discussion

The management of recurrent melanoma remains challenging. This patient with prolonged but localized in-transit melanoma showed remarkably improved clinical outcome when intralesional IL-2 was added to the interventions. The synergistic effect of IL-2 with V-beam laser is supported by animal models that show enhanced antitumor immunity with judicious combination of immunologic stimulation and laser devitalization of tumor nodules [2]. The benefits of systemic IL-2 are offset by its toxicities, limiting its usage to selected patients with advanced metastatic melanoma. We demonstrated that intralesional administration of IL-2 in combination with V-beam laser achieved rapid regression of in-transit metastasis with limited adverse effects. 

IL-2 induced vascular leakage is advantageous in localized treatment because local edema may initiate tumor necrosis that stimulates the immune response. Furthermore, intralesional injection of IL-2 achieves much higher local concentration and limits systemic adverse effects. Therefore a greater therapeutic effect could be expected. Indeed, studies indicated that intralesional IL-2 therapy was more effective than systemic, perilesional, and distant site IL-2 treatments [3]. One report showed that a complete local response was achieved in 69% of metastasized melanoma patients (33/48) by intratumorally injection of IL-2 [4].

Preferential absorption of laser radiance by hemoglobin and the subsequent conversion of light energy into thermal energy may cause blood vessel coagulation [5], which may enhance the local edematous and ischemic effect of IL-2. In addition, cancer cells are more vulnerable than normal cells to hyperthermia-induced cellular damage [6]. Considerable improvement in clinical outcome has been reported after hyperthermic therapy in tumors of many types, including melanoma [6]. Figure 1B summarizes the possible synergetic effect of intralesional IL-2 and V-beam therapy. 

## 4. Conclusions

Our results indicate that this combined therapy is a promising strategy for the treatment of in-transit metastatic melanoma. Further study in a larger patient population is warranted.
